# Effect of β-Alanine Metabolite on Gut Integrity and Immunity in Commercial Broiler Chickens Infected with *Eimeria maxima*

**DOI:** 10.3390/ani14172558

**Published:** 2024-09-03

**Authors:** Inkyung Park, Hyoyoun Nam, Youngsub Lee, Alexandra Smith, Thomas Rehberger, Hyun Lillehoj

**Affiliations:** 1 Animal Bioscience and Biotechnology Laboratory, Beltsville Agricultural Research Center, Agricultural Research Service-USDA, Beltsville, MD 20705, USAhyoyoun.nam@usda.gov (H.N.); youngsub.lee@usda.gov (Y.L.); 2 Arm & Hammer Animal and Food Production, Waukesha, WI 53186, USA; xandra.smith@churchdwight.com (A.S.); tomrehberger@gmail.com (T.R.)

**Keywords:** antibiotic alternative feed additives, gut health, gut metabolite, β-alanine, in vitro test

## Abstract

**Simple Summary:**

In a gut metabolomics analysis of growth-promoting probiotics which enhanced broiler growth, we discovered that β-alanine was significantly elevated as a gut metabolite. Furthermore, β-alanine is known to be synthesized into carnosine or have various biological functions in the body on its own. For these reasons, we evaluated the value of β-alanine as a new feed additive for *Eimeria*-infected broilers through this study, and our results will serve as a good example of utilizing metabolites revealed through metabolomics.

**Abstract:**

(1) Background: In a metabolomics analysis conducted to investigate the mechanisms behind the growth-promoting effects of probiotics in broilers, β-alanine was found to be significantly elevated. This led to the hypothesis that β-alanine could also contribute to growth-promoting effects in infected broilers. (2) Methods: An in vitro culture system was developed to assess β-alanine’s impact on proinflammatory cytokine response in chicken macrophage cells, gut integrity in chicken intestinal epithelial cells, and muscle differentiation in quail muscle cells and primary chicken embryonic muscle cells. In vivo animal feeding studies were then conducted to investigate the effects of dietary β-alanine on various disease parameters in *Eimeria maxima*-infected broiler chickens. (3) Results: In vitro, β-alanine treatment significantly decreased the gene expression of cytokines in chicken macrophage cells and increased occuldin expression in chicken intestinal epithelial cells. Dietary β-alanine increased the body weight of chickens following *Eimeria maxima* infection in the H-ALA group. Dietary β-alanine also suppressed cytokines and increased JAM-2 and occludin expression in the H-ALA group compared to the infected group without β-alanine supplementation. (4) Conclusions: These results strongly support the positive effects of dietary β-alanine on intestinal immune responses and gut barrier function in broiler chickens infected with *Eimeria maxima*.

## 1. Introduction

With increasing information and understanding of how complex and critical the gut ecosystem is for efficient animal growth, novel feed additives that can enhance growth efficiency and reduce our reliance on antibiotics with a primary focus on disease prevention rather than treatment will be desirable for increasing demand for food production [1]. Ultimately, how to protect chickens against external infections to maintain good gut health in commercial poultry production conditions against common parasites such as *Eimeria* spp. is a big challenge that needs to be addressed using various feed additives that can benefit microbial and host interaction [2,3]. Accordingly, there has been considerable interest in learning about the physiological changes induced by feed additives [3,4,5]. As a result, research on the digestion and absorption of feed nutrients, which are the direct causes of poultry growth, has been conducted, and there has been considerable scientific progress in understanding the effects of feed nutrients on poultry growth and immunity [4,6]. However, due to the complex physiological factors affecting gut health, a wider range of scientific knowledge is needed to understand and control the fundamental metabolic mechanisms associated with poultry growth or immunity [7,8]. Genomics is becoming widely applied to provide systemic information on microbial populations in the intestine [9]. However, it is impossible to understand the physiological functions of individual strains of more than 3 trillion intestinal microbes based on the results of genomics, and it is not easy to artificially control specific bacteria to dominate [10] in the gut microbiota community. However, understanding the function of gut metabolites that are produced by intestinal microbes may provide considerable knowledge [11,12,13] that can lead to better utilization of gut microbe-produced metabolites in modulating host physiological responses. The estimated number of metabolites in the intestine obtained from global metabolomics is within 1000, and these metabolites closely interact with various receptors responsible for nutrient absorption, immune surveillance, and gut permeability in the brush border area in the host intestinal epithelium as pathogen-associated molecular patterns [14,15,16].

In our previous studies, we used two different strains of growth-promoting probiotic *Bacillus subtilis* in commercial broiler chickens infected with *Eimeria maxima* to mitigate gut damage, inflammatory response, and growth reduction successfully [17]. To better understand the underlying interaction between the gut microbiota and coccidiosis-induced physiological changes, we conducted a systemic analysis of metabolites through global metabolomics. As a result, a total of 674 metabolites were identified, including 11 dipeptides made up of two amino acids [18]. Among the dipeptides, carnosine increased 4.38 times compared to the control group, and interestingly, β-alanine, an amino acid, also showed a significant increase, 3.18 times above the control (unpublished data). Carnosine, composed of β-alanine and histidine, is produced by ingested food or intestinal microorganisms and absorbed into the intestine and throughout the body. In this process, it has been reported that carnosine itself or β-alanine and histidine perform their physiological functions via the degradation or synthesis enzymes present in the body. Carnosine is generally known to mainly maintain pH balance, reduce oxidative stress in the body, and help muscle formation [19,20,21]. For the purpose of enhancing the physiological functions of carnosine, β-alanine was fed to animals to show improved results in muscle growth and antioxidant activity [22,23].

The aim of this study was to better understand the interaction of the gut microbiota and their metabolites in influencing host physiological response and the beneficial effects of dietary β-alanine as a feed additive in commercial poultry production. In particular, the study examined (1) how β-alanine affects the host’s innate immune response in chicken macrophage cells (CMCs), as well as its impact on barrier function in chicken intestinal epithelial cells (IECs), anticoccidial activity against *Eimeria maxima* (*E. maxima*) sporozoites, and muscle cell differentiation in quail muscle cells (QMCs) and primary chicken embryonic muscle cells (PMCs) in vitro, and (2) how dietary β-alanine influences growth performance, intestinal immunity, epithelial integrity, and nutrient transporters in young broiler chickens infected with *E. maxima*. These investigations will shed light on the role of the gut microbiota and the various underlying mechanisms through which various beneficial gut bacteria exert beneficial physiological changes in the host.

## 2. Materials and Methods

All experiments that were related to animals were approved by the Beltsville Agricultural Research Center Institutional Animal Care and Use Committee (#19–018).

### 2.1. Experiment 1: In Vitro Study

#### 2.1.1. Anticoccidial Assay

The direct effect of β-alanine on Eimeria maxima sporozoites was assessed using an in vitro sporozoite viability assay, following methods previously described [24,25]. In brief, fresh sporulated oocysts of Eimeria maxima were placed in a 2 mL microcentrifuge tube and disrupted using 0.5 mm glass beads using a Mini-Bead beater (BioSpec Products, Bartlesville, OK, USA) for 10 s. The released sporocysts were washed with chilled Hanks’ balanced salt solution (HBSS, Hyclone, Logan, UT, USA) and incubated with 10 mL of excystation media (0.25% trypsin and 0.014 M taurocholic acid, pH 7.4) at 41 °C for 1 h to release sporozoites. The sporozoites (2.5 × 10^5^) were then seeded into each well of a 96-well plate. Chicken NK-lysin peptide (Genscript, Piscataway, NJ, USA) was used as a positive control at concentrations of 1.0, 10, and 100 µg/mL. β-alanine was tested at low (0.1 µg/mL), medium (1.0 µg/mL), and high (10 µg/mL) concentrations. Freshly prepared live sporozoites were treated with β-alanine and incubated at 41 °C for 3 h. A fluorescent dye (AO/PI staining solution, Nexcelom Bioscience LLC, Lawrence, MA, USA) was added to each well at a 1:1 ratio, and the number of live sporozoites was quantified using a Cellometer (Nexcelom Bioscience). All experiments were conducted independently at least three times.

#### 2.1.2. Culture of Epithelial Chicken Cell Line (IEC) and Chicken Macrophage Cell Line (CMC)

IEC (MM-CHiC clone, 8E11, MicroMol GmbH, Karlsruhe, Germany) and CMC (chicken macrophage-like line, HD11, MicroMol GmbH) were seeded at a density of 2 × 10^5^ cells/mL in 24-well plates and cultured in Dulbecco’s modified Eagle medium (DMEM)/F-12 (Hyclone) containing 10% heat-inactivated fetal bovine serum (FBS, Hyclone) and 1% antibiotic solution [penicillin (10,000 unit/mL) and streptomycin (10 mg/mL), Gibco, Grand Island, NY, USA]. The cells were incubated at 41 °C in a humidified atmosphere with 5% CO_2_ for 24 h to allow for cell adhesion. After this period, lipopolysaccharide (LPS, Sigma-Aldrich, St. Louis, MO, USA) was added to each well at a concentration of 1.0 µg/mL, along with β-alanine (Sigma-Aldrich) at concentrations of 0.0, 0.1, 1.0, and 10.0 µg/mL. Following an additional 18 h incubation, the cells were harvested using lysis buffer (Qiagen, Valencia, CA, USA) and 2-mercaptoethanol (Sigma-Aldrich). RNA was extracted from the IEC and the CMC using the RNeasy Isolation Kit (Qiagen) on a QIAcube (Qiagen), and gene expression was analyzed using quantitative real-time polymerase chain reaction (qRT-PCR). All experiments were conducted in triplicate.

#### 2.1.3. Quail Muscle Cell (QMC) Culture

The QMCs (2 × 10^5^/mL) were seeded in 24-well plates following previously described methods [26,27]. The cells were cultured in Medium 199 (Hyclone) with 10% FBS and 1% antibiotic solution until they reached 70% confluency. For 12 wells, the media were replaced with Medium 199 containing 0.5% FBS and 1% antibiotic solution to induce cell differentiation. For the remaining 12 wells, the media were replaced with basic Medium 199 containing 10% FBS and 1% antibiotic solution to maintain cell proliferation. β-alanine was added to each well at concentrations of 0.0, 0.1, 1.0, and 10.0 µg/mL. After incubation at 41 °C in a humidified atmosphere with 5% CO_2_ for 18 h, the cells were harvested using lysis buffer and 2-mercaptoethanol. RNA was extracted from the QMCs using the RNeasy Isolation Kit on a QIAcube, and gene expression was analyzed using qRT-PCR. All experiments were conducted independently at least three times.

#### 2.1.4. Primary Chicken Embryonic Muscle Cell (PMC) Culture

Eggs for the embryonic muscle cell culture were purchased from Moyer’s hatchery (Quakertown, PA, USA). The PMC culture protocol was modified from a method described previously [27,28]. In brief, the eggs were incubated at 41 °C and 70% humidity. The pectoralis major region of 13-day-old embryos was extracted, minced, and digested with 0.05% trypsin-EDTA (Sigma-Aldrich) at 37 °C for 20 min. The PMCs were washed 2–3 times with HBSS and seeded at a density of 2 × 10^5^ cells/mL in 24-well plates. The cells were maintained in DMEM (Hyclone) containing 10% FBS and 1% antibiotic solution until they reached 70% confluency. For 12 wells, the medium was replaced with DMEM with 2% FBS and 1% antibiotic solution to induce cell differentiation, while for the remaining 12 wells, the medium was replaced with DMEM containing 10% FBS and 1% antibiotic solution to maintain cell proliferation. β-alanine was added to each well at concentrations of 0.0, 0.1, 1.0, and 10.0 µg/mL. After incubation at 41 °C in a humidified atmosphere containing 5% CO_2_ for 18 h, the cells were harvested using lysis buffer and 2-mercaptoethanol. RNA was extracted from the PMCs using the RNeasy Isolation Kit on a QIAcube and eluted in 30 μL RNase-free water. Gene expression was analyzed using qRT-PCR. All experiments were performed independently at least three times.

#### 2.1.5. Reverse Transcription for PCR

RNA quantity was measured using a NanoDrop (ND-1000) spectrophotometer (NanoDrop Products, Wilmington, DE, USA) by assessing absorbance at 260 nm. RNA purity was determined by calculating the OD260/OD280 ratio. A total of 1 µg RNA was reverse transcribed to cDNA using the QuantiTect^®^ reverse transcription kit (Qiagen). In brief, the RNA samples were treated with genomic DNA wipeout buffer (Qiagen) at 42 °C for 2 min to eliminate any genomic DNA contamination. Reverse transcription (RT) was conducted by adding Quantiscript Reverse Transcriptase, Quantiscript RT buffer, and RT primer mix to the genomic DNA-free samples. The reaction was carried out in a thermal cycler (Mastercycler^®^ EP Gradient S; Eppendorf, Hauppauge, NY, USA) with the following cycling parameters: 42 °C for 30 min, followed by inactivation of reverse transcriptase at 95 °C for 3 min. Aliquots of cDNA samples were stored at −20 °C.

#### 2.1.6. Analysis of Cytokines, Tight Junction Proteins, and Muscle Cell Growth Markers Using qRT-PCR

Tight junction (TJ) protein (occludin, ZO-1) and mucine (MUC2) were examined using qRT-PCR with RNA samples extracted from the IECs. Proinflammatory cytokine levels (IL-1β, IL-6, and IL-8) were measured in the CMCs using the extracted RNA samples. Proliferation and differentiation markers of muscle cells (*Pax7* and *MyoG*) were determined using RNA samples obtained from the QMCs and PMCs. qRT-PCR was conducted on an Agilent Mx3000 P QPCR System (Agilent Technologies, Santa Clara, CA, USA) and Brilliant SYBR Green qRT-PCR Master Mix (Stratagene, Thermo Fisher Scientific, Waltham, MA, USA). Oligonucleotide primer sequences and product size used for qRT-PCR are provided in Table 1. A melting curve was generated at the end of each run to confirm the presence of a single amplification product without primer dimers. Standard curves were produced using serial 5-fold dilutions of cDNA. The fold changes in transcript levels were normalized to glyceraldehyde-3-phosphate dehydrogenase and are relative to the transcript expression in the unstimulated control group (normalized to 1) using the comparative 2^−ΔΔCt^ method as previously described [29].

### 2.2. Experiment 2: In Vivo Study

#### 2.2.1. Chickens and Experimental Design

**Figure 1:**
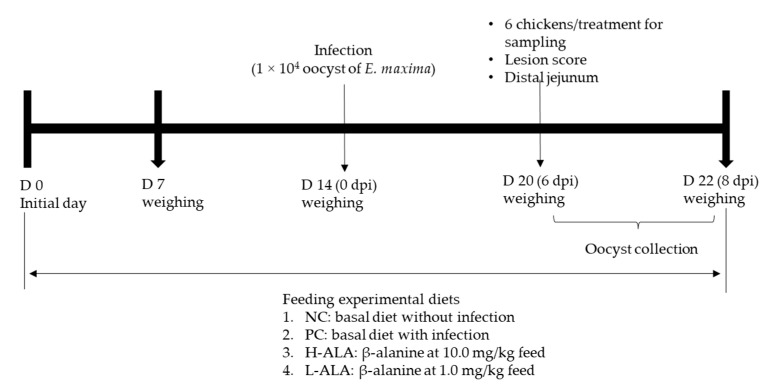
Experimental design outline for experiment 2. dpi: day post infection.

A total of 120 newly hatched male broiler chickens (Ross 708) were obtained from Longenecker’s hatchery (Elizabethtown, PA, USA) at 0 days of age. Upon arrival at the Beltsville ARS facility, the chickens were weighed and assigned to four dietary treatments in a randomized complete block design, with initial body weight (BW) as the block factor (heavy and light). The dietary treatments included a corn- and soybean meal-based basal diet (Table 2) for non-infected chickens (NC), a basal diet for *E. maxima*-infected chickens (PC), and PC supplemented with β-alanine at 10.0 mg/kg feed (H-ALA) and 1.0 mg/kg feed (L-ALA). Crystalline β-alanine was sourced from Sigma-Aldrich. Each treatment group was housed in six wire-bottom cages, with five chickens per cage (0.65 × 0.75 m^2^). The chickens had ad libitum access to water and feed throughout the experimental period. A schematic representation of the experimental design is provided in Figure 1.

#### 2.2.2. Determination of Body Weight 

The chickens’ BW was measured on d 0, 7, 14, 20, and 22 to calculate average daily gain (ADG). Deceased chickens were removed and weighed to adjust growth data. 

#### 2.2.3. Oral Infection with *E. maxima*

All chickens, except those in the NC group, were orally infected with *E. maxima* (1.0 × 10^4^ sporulated oocysts/chicken, Beltsville strain 41A) on day 14 using methods previously described [17]. The ratios of sporulated oocysts were 87.5 ± 2.1%. The purity of the *E. maxima* infection was confirmed via DNA genotyping [31].

#### 2.2.4. Collection of Intestinal Samples

One chicken with an average BW from each cage was euthanized by cervical dislocation on d 20, and their intestines were removed for further analysis. A small section (2 cm) of the distal jejunum was collected from each intestine and to remove the digesta, it was gently rinsed with saline and then stored in RNAlater^®^ (Invitrogen, Carlsbad, CA, USA) at −20 °C until further analysis.

#### 2.2.5. Jejunal Lesion Scoring

Jejunal lesion scoring was conducted on a 15 cm long distal jejunum sample on d 20. Four independent observers graded the intestinal lesions on a discrete ordinal scale of 0 (none) to 4 (high) in a blinded manner, following previously described methods [32].

#### 2.2.6. Fecal Oocyst Shedding

Non-absorbent coated paper was placed under each cage on day 20, and the entire fecal samples on the paper were collected from d 20 to 22 (6 to 8 days post infection: dpi). The number of oocysts was counted using the McMaster chamber according to previously described protocols [27]. The following formula was used for calculating the total oocyst per chicken: 

Total oocysts/chicken = [oocyst count × dilution factor × (fecal sample volume/counting chamber volume)]/number of chickens per cage.

#### 2.2.7. RNA Isolation and Reverse Transcription from Jejunal Samples

Total RNA was extracted from jejunal samples preserved in RNAlater^®^ following the manufacturer’s guidelines. Approximately 50 mg of the jejunal tissue was homogenized in 1 mL of TRIzol (Invitrogen) using a handheld homogenizer (TissueRuptor; Qiagen). Chloroform was added to the homogenized samples, followed by centrifugation at 12,000× *g* for 15 min at 4 °C to separate the phases. RNA in the colorless upper aqueous phase was precipitated using 100% isopropanol (Sigma-Aldrich). The RNA pellet was washed with 75% ethanol (Sigma-Aldrich), air-dried, and resuspended in RNase-free water (Invitrogen). RNA quantity was assessed using a NanoDrop (ND-1000) spectrophotometer (NanoDrop Products) based on absorbance at 260 nm. RNA purity was assessed by calculating the OD260/OD280 ratio. Total RNA (1 µg) was reverse transcribed to cDNA using the QuantiTect^®^ reverse transcription kit (Qiagen). In brief, the RNA samples were incubated with genomic DNA wipeout buffer at 42 °C for 2 min to remove any genomic DNA contamination. Reverse transcription (RT) was performed by adding Quantiscript Reverse Transcriptase, Quantiscript RT buffer, and RT primer mix to the genomic DNA-depleted samples. The reaction was conducted in a thermal cycler (Mastercycler^®^ EP Gradient S) with the following conditions: 42 °C for 30 min, followed by reverse transcriptase inactivation at 95 °C for 3 min. Aliquots of the cDNA samples were stored at −20 °C. 

#### 2.2.8. Gene Expression Analysis via qRT-PCR 

The sequences of oligonucleotide primer used for qRT-PCR are listed in Table 1. The expression of various cytokines, TJ proteins, and nutrient transporters in the jejunum was assessed, including cytokines (IL-1β, IL-6, IL-8, IL-10, IFN-γ, and TNFSF15), TJ proteins (claudin-1, claudin-2, JAM-2, occludin, ZO-1, and ZO-2), and nutrient transporters (BAT1, B0AT1, CAT1, EAAT, GLUT1, GLUT2, GLUT5, LAT1, LAT2, and SGLT). Glyceraldehyde-3-phosphate dehydrogenase was used as the reference gene. Amplification and detection were performed using the Stratagene Mx3000P qPCR system (Agilent Technologies Inc.) and RT2 SYBR Green qPCR master mix (Qiagen). Each sample was analyzed in triplicate, and non-specific primer amplification was evaluated using no-template controls. Standard curves were generated using log10 diluted RNA standards, and the transcript levels were normalized to those of glyceraldehyde-3-phosphate dehydrogenase using the Q-gene program [27].

#### 2.2.9. Statistical Analysis

In vitro data were analyzed using a PROC GLM in SAS version 9.4 (SAS Inc., Cary, NC, USA). A *p*-value less than 0.05 was considered significant. Data were accepted when skewness and kurtosis ranged in values at ±3 and ±7, respectively. In vivo data were analyzed using a mixed model (PROC MIXED) in SAS, with each cage considered as an experimental unit. The results are presented as least squares mean values with the pooled standard error of the mean. Probability values less than 0.05 were considered significant. When the overall effect was significant, mean values were compared pairwise using the PDIFF option.

## 3. Results

### 3.1. Experiment 1

#### 3.1.1. Anti-Coccidial Activity against Sporozoites of *E. maxima*

**Figure 2:**
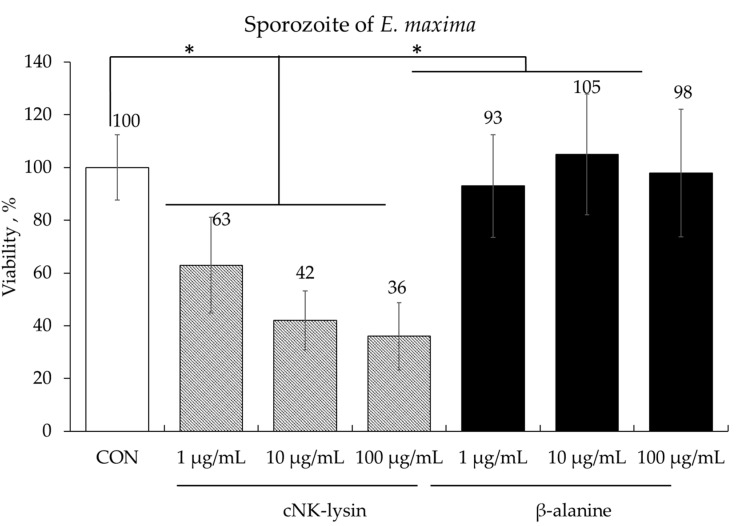
Anticoccidial effect of β-alanine on *Eimeria maxima* sporozoites. Each bar represents the mean ± SEM (*n* = 3). The sporozoite concentration in the control (CON) group was 2.5 × 10^5^ sporozoites/mL. Significance was determined using a PROC GLM in SAS. A *p*-value less than 0.05 (*) was considered significant.

Treatment of freshly prepared *E. maxima* sporozoites with cNK-lysin, used as a positive control, a dose-dependent killing effect, ranging from 37% to 64%, compared to the control (CON) group (*p* < 0.05). In contrast, no significant differences were observed in sporozoite number between the groups treated with various doses of β-alanine and the CON group (Figure 2).

#### 3.1.2. Effects of β-Alanine on Chicken Intestinal Epithelial Cells and Chicken Macrophage Cells

**Figure 3:**
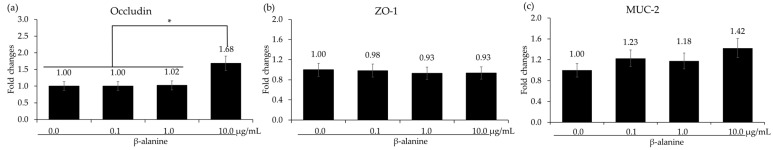
mRNA expression level of tight junction proteins and mucin in chicken epithelial cells treated with β-alanine. Each bar represents the mean ± SEM (*n* = 3). Transcript levels were measured using quantitative RT-PCR and normalized to GAPDH transcript levels. Significance was determined using PROC GLM in SAS, with a *p*-value less than 0.05 (*) considered significant.

**Figure 4:**
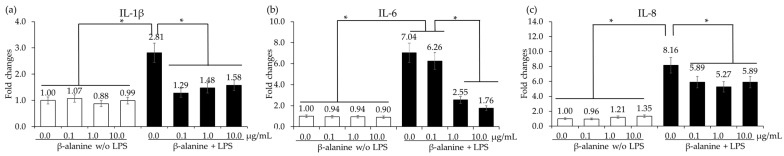
Secretion of proinflammatory cytokines in chicken macrophages treated with LPS and β-alanine. Each bar represents the mean ± SEM (*n* = 3). Cytokine transcript levels were measured using quantitative RT-PCR and normalized to GAPDH levels. Significant results are indicated by: * = *p* < 0.05.

In vitro treatment of chicken IECs and CMCs with β-alanine at 10.0 µg/mL significantly increased (*p* < 0.05) the gene expression of occludin compared to the other treatment groups. However, there were no significant differences (*p* > 0.05) observed in the gene expression of ZO-1 and MUC-2 among the treatment groups (Figure 3). The presence of β-alanine without LPS did not induce any significant changes (*p* > 0.05) in cytokine levels in CMCs, regardless of the β-alanine concentrations. However, when the CMCs were treated with LPS, there was a significant increase (*p* < 0.05) in all the cytokine levels. In the LPS-treated group, treatment with β-alanine significantly decreased (*p* < 0.05) the gene expression levels of IL-1β, IL-6, and IL-8 in all treatment groups except for IL-6, which was treated with 0.1 µg of β-alanine (Figure 4).

#### 3.1.3. Effects of β-Alanine on the Proliferation and Differentiation of Quail Muscle Cells and Primary Chicken Embryonic Muscle Cells

**Figure 5:**
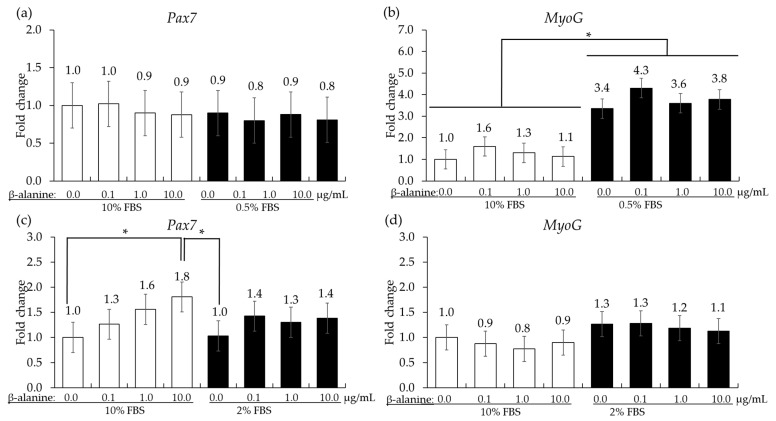
Proliferation and differentiation of quail muscle cells (**a**,**b**) and chicken embryo muscle cells (**c**,**d**) influenced by FBS concentration and β-alanine. Each bar represents the mean ± SEM (*n* = 3). Transcript levels were measured using quantitative RT-PCR and normalized to GAPDH transcript levels. Significant results are indicated by: * = *p* < 0.05.

In QMCs, reducing the FBS concentration from 10% to 0.5% did not change the gene expression level of *Pax7* but significantly increased (*p* < 0.05) the gene expression level of *MyoG* irrespective of β-alanine administration. In PMCs, the administration of β-alanine resulted in a dose-dependent increase (*p* < 0.05) in *Pax7* levels in the presence of 10% FBS. However, in all treatment groups cultured in 2% FBS, no significant changes (*p* > 0.05) were observed in *Pax7* levels. The gene expression of *MyoG* showed (*p* > 0.05) no significant changes in either 10% or 2% FBS (Figure 5).

### 3.2. Experiment 2

#### 3.2.1. Growth of Chickens

The chickens used in this animal trial were allocated into experimental cages based on their BW to ensure no difference (*p* = 1.000) in their BWs among the treatment groups (Table 3). Up until day 14, which is before infection, dietary β-alanine supplementation did not show (*p* > 0.05) any change in BWs compared to the control group (NC). The *E. maxima* infection on day 14 decreased (*p* < 0.001) the BW of the chickens on d 20 [6 dpi; 860 (NC) to 748 g (average of infected groups)] and d 22 (8 dpi; 1017 to 815 g) compared to the NC, regardless of the supplementation of β-alanine. Dietary β-alanine supplementation during the infection period did not change the BW of chickens at 6 dpi compared to the NC but both H-ALA and L-ALA groups showed increased BW at 8 dpi compared to the PC group. Dietary supplementation of β-alanine during the infection period did not affect the BW of chickens at 6 dpi when compared to the infected control group (PC). However, both H-ALA (763 to 843 g) and L-ALA (763 to 839 g) groups showed (*p* < 0.001) significantly increased BW at 8 dpi compared to the PC. ADG was the same as BW with no changes (*p* > 0.05) compared to NC prior to infection. *E. maxima* infection decreased (*p* < 0.001; 69.4 vs. 38.2 g) the ADG of chickens compared to NC regardless of β-alanine supplementation, and its effect lasted until the end of the experiment. Throughout the entire infection period, both H-ALA (*p* < 0.001; 38.2 to 47.3 g) and L-ALA (*p* = 0.035; 38.2 to 43.3 g) groups showed significantly increased ADG compared to the PC group, and there was no difference between H-ALA and L-ALA.

#### 3.2.2. Intestinal Lesion Scores and Fecal Oocyst Shedding

**Figure 6:**
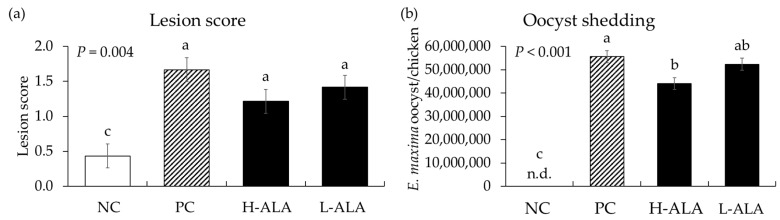
Lesion score and oocyst shedding in chickens fed a β-alanine supplemented diet during infection with *Eimeria maxima*. NC: basal diet, PC: basal diet for infected chickens, H-ALA: β-alanine at 10.0 mg/kg feed, L-ALA: β-alanine at 1.0 mg/kg feed, n.d.: not detected. All chickens, except NC, were infected by oral gavage at day 14 with 1.0 × 10^4^ oocysts/chicken of *E. maxima*. ^a~c^ Bars with different letters indicate significant differences (*p* < 0.05). Each bar represents the mean ± SEM (*n* = 6). Lesion score was collected from distal jejunal tissue on day 20 (6 dpi: days post infection), and fecal samples were collected from 6 to 8 dpi to calculate oocyst shedding.

*E. maxima* infection significantly increased (*p* = 0.004) intestinal lesion scores [0.4 to 1.43 (average of infected groups)] in the distal jejunum regardless of β-alanine supplementation. The score of H-ALA was decreased (*p* < 0.001; 1.67 to 1.22) compared to that of PC (Figure 6a). Similar to gut lesion scores, the number of *E. maxima* oocysts measured in fecal samples collected from 6 dpi to 8 dpi significantly increased (*p* < 0.001; 0 to 5.1 × 10^7^) due to *E. maxima* infection compared to NC. Among the infected treatments, the H-ALA group showed significantly reduced (*p* < 0.05; 5.6 × 10^7^ to 4.4 × 10^7^) fecal oocysts compared to PC (Figure 6b).

#### 3.2.3. Pro-Inflammatory Cytokines

**Figure 7:**
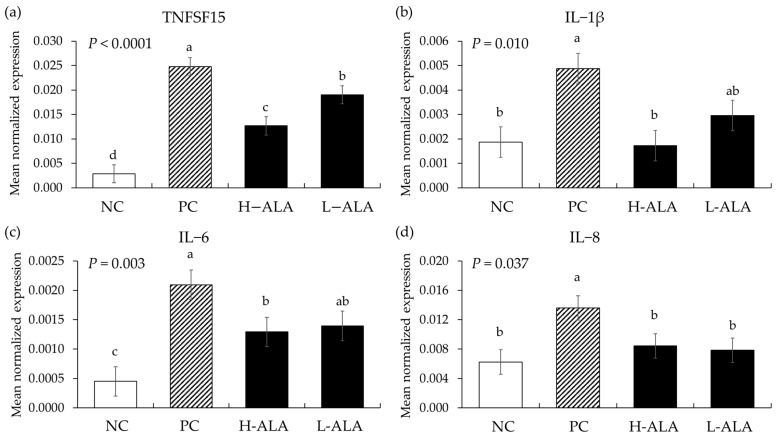
Transcripts of proinflammatory cytokines in the jejunum of chickens fed a β-alanine supplemented diet during *Eimeria maxima* infection in experiment 2. NC: basal diet, PC: basal diet for infected chickens, H-ALA: β-alanine at 10.0 mg/kg feed, L-ALA: β-alanine at 1.0 mg/kg feed, all chickens, except NC, were infected by oral gavage at day 14 with 1.0 × 10^4^ oocysts/chicken of *E. maxima*. ^a~d^ Bars with different letters indicate significant differences (*p* < 0.05). Each bar represents the mean ± SEM (*n* = 6). Data were collected on day 20 (6 dpi: days post infection). Cytokine transcript levels were measured using quantitative RT-PCR and normalized to GAPDH levels.

*E. maxima* infection (PC) without β-alanine supplementation increased the gene expression of TNFSF15 (*p* < 0.0001; 2.9 × 10^−3^ to 2.5 × 10^−2^), IL-1β (*p* = 0.004; 1.9 × 10^−3^ to 4.9 × 10^−3^), IL-6 (*p* = 0.00; 4.5 × 10^−4^ to 2.1 × 10^−3^), and IL-8 (*p* = 0.007; 6.3 × 10^−3^ to 1.4 × 10^−2^) in the distal jejunum compared to the NCs (Figure 7) whereas H-ALA suppressed the releases of TNFSF15 (*p* = 0.0003; 2.5 × 10^−2^ to 1.3 × 10^−2^), IL-1β (*p* = 0.0028; 4.9 × 10^−3^ to 1.7 × 10^−3^), IL-6 (*p* = 0.040; 2.1 × 10^−3^ to 1.3 × 10^−3^), and IL-8 (*p* = 0.043; 1.4 × 10^−2^ to 8.4 × 10^−3^) compared to the correspondent cytokines of the PCs. L-ALA also suppressed the releases of TNFSF15 (*p* = 0.0003; 2.5 × 10^−2^ to 1.9 × 10^−2^) and IL-8 (*p* = 0.027; 1.4 × 10^−2^ to 7.9 × 10^−3^) compared to those cytokines of PCs. In the case of TNFSF15, that (1.3 × 10^−2^) of H-ALA significantly decreased (*p* = 0.029) compared to that (1.9 × 10^−2^) of L-ALA. 

#### 3.2.4. Th1 Cytokines

**Figure 8:**
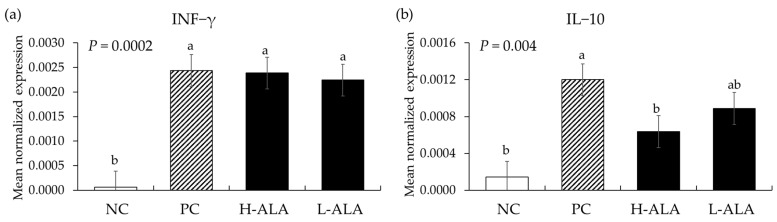
Transcripts of Th1 cytokines in the jejunum of chickens fed a β-alanine supplemented diet during *Eimeria maxima* infection in experiment 2. NC: basal diet, PC: basal diet for infected chickens, H-ALA: β-alanine at 10.0 mg/kg feed, L-ALA: β-alanine at 1.0 mg/kg feed, all chickens, except NC, were infected by oral gavage at day 14 with 1.0 × 10^4^ oocysts/chicken of *E. maxima*. ^a~b^ Bars with different letters indicate significant differences (*p* < 0.05). Each bar represents the mean ± SEM (*n* = 6). The data were collected on day 20 (6 dpi: days post infection). Cytokine transcript levels were measured using quantitative RT-PCR and normalized to GAPDH levels.

*E. maxima* infection increased (*p* < 0.0003) the gene expression of IFN-γ (from 6.2 × 10^−5^ to an average of 2.3 × 10^−3^) regardless of dietary β-alanine supplementation when compared to NC (Figure 8). Similarly, *E. maxima* infection significantly increased (*p* = 0.0006) the IL-10 level (from 1.4 × 10^−4^ to 1.2 × 10^−3^) in the distal jejunum of chickens compared to the NC. However, H-ALA significantly decreased (*p* = 0.036) the gene expression level of IL-10 (from 1.2 × 10^−3^ to 6.4 × 10^−4^) compared to that of the PC.

#### 3.2.5. Tight Junction Proteins

**Figure 9:**
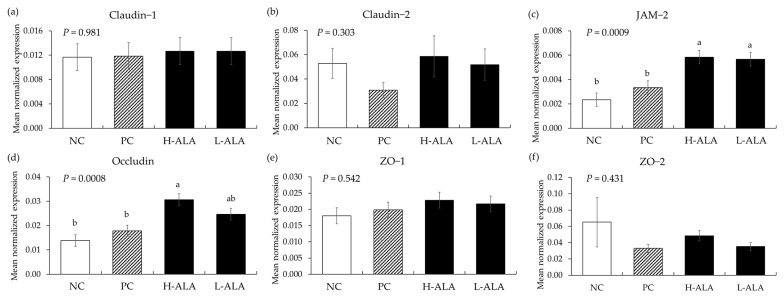
Transcripts of tight junction proteins in the jejunum of chickens fed a β-alanine supplemented diet during *Eimeria maxima* infection in experiment 2. NC: basal diet, PC: basal diet for infected chickens, H-ALA: β-alanine at 10.0 mg/kg feed, L-ALA: β-alanine at 1.0 mg/kg feed, all chickens, except NC, were infected by oral gavage at day 14 with 1.0 × 10^4^ oocysts/chicken of *E. maxima*. ^a~b^ Bars with different letters indicate significant differences (*p* < 0.05). Each bar represents the mean ± SEM (*n* = 6). The data were collected on day 20 (6 dpi: days post infection). Transcript levels were measured using quantitative RT-PCR and normalized to GAPDH levels.

*E. maxima* infection (PC) without β-alanine did not affect (*p* > 0.05) the gene expression of tight junction proteins in the distal jejunum of chickens compared to the NC which were fed the basal diet (Figure 9). Additionally, dietary β-alanine supplementation regardless of its dose did not influence (*p* > 0.05) the gene expression levels of claudins and ZOs in the distal jejunum compared to that of the NC and PC. However, H-ALA increased the gene expression of JAM-2 (*p* = 0.001; from 2.3 × 10^−3^ to 5.8 × 10^−3^) and occludin (*p* = 0.0002; from 1.4 × 10^−2^ to 3.1 × 10^−2^) compared to the NC and PC. L-ALA also increased (*p* = 0.001) the JAM-2 level (from 2.3 × 10^−3^ to 5.7 × 10^−3^) of distal jejunum compared to the NC and PC. 

#### 3.2.6. Nutrient Transporters

*E. maxima* infection (PC) significantly decreased (*p* > 0.05) the expression of BAT1 (from 0.43 to 0.13), B0AT1 (from 0.074 to 0.027), GLUT1 (from 0.0057 to 0.0013), GLUT2 (from 0.022 to 0.007), GLU5 (from 0.073 to 0.016), LAT1 (from 0.07 to 0.015), and LAT2 (from 0.0072 to 0.0014) expression levels compared to those of the NC (Table 4). However, L-ALA significantly increased (*p* < 0.05) the gene expression levels of BAT1 (from 0.13 to 0.29), B0AT1 (from 0.027 to 0.053), LAT1, and LAT2 in the jejunum of chickens compared to the PC. L-ALA did not change (*p* > 0.05) any nutrient transport compared to the PC.

## 4. Discussion

In the current study, we used various in vitro experiments that will guide the planning of the minimum number of animal trials required to reduce the use of live animals in animal experimentation. The most important goal of designing these in vitro studies was to evaluate the dietary effects of β-alanine in host physiological responses that would predict outcomes in terms of growth, gut health, and host immunity when young broiler chickens treated with β-alanine were infected with intracellular intestinal parasites, *E. maxima*. First, we investigated the direct effect of β-alanine on the sporozoite of *E. maxima*. As a positive control, cNK-lysin, a chicken homolog to human granulysin with powerful antimicrobial activity on *Eimeria* sporozoites [24,29,33], was used. In vivo animal trials using an established coccidiosis disease model showed that cNK-lysin killed freshly prepared *Eimeria* sporozoites in a dose-dependent manner whereas β-alanine did not have any direct effect on sporozoite viability.

In vitro treatment of chicken epithelial cells with β-alanine at 10 µg/mL significantly increased the gene expression level of occludin by about 68% compared to other treatments. Although it is not well known how β-alanine increases occludin expression in chicken intestinal epithelial cells, a previous study [34] suggested that β-alanine may promote protein synthesis in cells to improve cell function. In vitro testing of β-alanine on the inflammatory response of chicken macrophages indicated significant suppression of LPS-induced IL-1β, IL-6, and IL-8 production upon β-alanine treatment. According to a systemic review with the Bayesian-based meta-analysis of all published aggregate data using a dose–response (Emax) model, β-alanine has been shown to increase the concentration of carnosine proportionally [35]. Furthermore, carnosine was shown to reduce the expression of IL-8 in Caco-2 cells stimulated by hydrogen peroxide or TNF-α [20]. Although we did not directly measure the concentration of carnosine in our study, our results imply that the decreased cytokine expression may be due to the increase in carnosine content. In the case of muscle cells, when the FBS concentration in the culture medium of QMCs was reduced from 10% to 0.5%, there was no change in the gene expression level of *Pax7*, a cell proliferation marker, although the *MyoG* expression level increased, indicating that these QMCs cycles were transitioning to the differentiation phase. In this state, the administration of β-alanine exerted no effect on the expression of these two genes. In the PMCs, despite reducing the FBS concentration from 10% to 2%, there was no change in the expression of *Pax7* or *MyoG*. Instead, an increase in *Pax7* level was observed when β-alanine was administered at 10 µg/mL under 10% FBS, suggesting that β-alanine could prolong the proliferation of PMCs. Observations of these muscle cell markers confirmed that even the same muscle cells can have different conditions depending on the type of cell. Contrary to our expectation that β-alanine administration would lead to an increase in *MyoG* expression related to the creation of new myotubes to affect muscle creation, β-alanine did not affect *MyoG* levels. In most research results, dietary supplementation of β-alanine led to an increase in carnosine content in muscles [36]. However, in several studies, the increase in carnosine did not lead to muscle cell growth [36]. The results of our in vitro study may suggest that β-alanine could be used as a potential feed additive for chickens infected with *E. maxima* to strengthen gut integrity through the upregulation of occludin expression, reducing the release of pro-inflammatory cytokine responses and by increasing the proliferation of PMCs. Based on these observations from in vitro studies, β-alanine was fed to chickens infected with *E. maxima* to evaluate its effect on *E. maxima* infection. Most of the research on β-alanine in the field of animal science is dominated by nutritional aspects and studies on carnosine. However, nutritional research on β-alanine and the increase in carnosine production in the body—essential for performing physiological functions such as antioxidant activity, stress reduction, and pH balance, all of which are closely linked to the host immune system—is necessary. Therefore, more immunological research is needed to better understand the basic mechanism of β-alanine in diseases like coccidiosis. 

Chickens when infected with *E. maxima* (10,000 sporulated oocyst) show an up to 25% reduction in BW compared to the uninfected control chickens. Among these infected chickens, dietary feeding with 10.0 mg and 1.0 mg of β-alanine per kg of feed led to an approximate 10% increase in their body weight. This result was also reflected in the ADG. Studies on the effect of dietary β-alanine on poultry growth are rare. Qi et al. [37] showed that dietary supplementation of broiler chickens with β-alanine from hatch to day 42 increased BW and ADG during the first half of the period (days 1–21), indicating improved FCR induced by β-alanine. In contrast, Lackner et al. [38] showed the absence of a β-alanine effect on BW or ADG at days 33 and 53 of broiler age. Both studies focused more on chickens’ muscle growth and other factors related to carnosine in muscle tissue than on the growth of broiler chickens in the absence of infection. In pigs, β-alanine at 0, 300, 600, and 1200 mg/kg was fed to weaned pigs for up to 28 days to investigate growth performance, inflammatory responses, intestinal permeability, and serum biochemical indices. Dietary β-alanine supplementation did not have any significant effects on growth performance, but it promoted the differentiation of intestinal cells, increased the turnover rate of new cells, and enhanced the regenerative ability of intestinal cells [39].

The beneficial effects of dietary β-alanine on enhancing BW and ADG in *E. maxima*-infected chickens as we have shown in the current experiment are very promising. Infection with *E. maxima*, which targets the chicken’s jejunum for invasion and intracellular development [40], significantly increased intestinal damage and gut lesion scores. However, the administration of 10 mg of β-alanine resulted in a 27% decrease in gut lesion score. In addition, between 6 and 8 dpi, an increase in the level of fecal oocyst output was observed due to *E. maxima* infection. Similar to the gut lesion scores, the administration of 10 mg of β-alanine resulted in a 22% decrease in oocysts in the intestines of chickens infected with *E. maxima*. One interesting finding is that β-alanine did not directly damage the oocysts of *E. maxima* in vitro despite its effect on oocyst number when it was fed to the chickens. The answer to this may be found in the analysis of the inflammatory response, tight junction proteins, and nutrient transporters located in the epithelium of the jejunum. Firstly, an analysis of host proinflammatory responses revealed that, as confirmed in our previous studies [27,41,42], the jejunal cytokines (TNFSF15, IL-1β, IL-6, and IL-8) of chickens infected with *E. maxima* in the current experiment were increased more than twofold compared to those uninfected control chickens. Regardless of dietary supplemental concentrations, two concentrations of β-alanine reduced the gene expression levels of TNFSF15 and IL-8, compared to the levels shown in *E. maxima*-infected chickens which were not supplemented with β-alanine. Furthermore, the dietary supplementation of young chickens with 10 mg of β-alanine suppressed IL-1β and IL-6. Regardless of concentration, dietary β-alanine supplementations reduced the cytokine levels of TNFSF15 and IL-8 compared to those of the NC group. INF-γ, which is linked to Th1 responses, was not reduced by β-alanine despite the increase caused by *E. maxima* infection. However, 10 mg of β-alanine suppressed the expression of IL-10 induced by *E. maxima* infection. These results support the notion that dietary β-alanine can decrease the pro-inflammatory cytokine response caused by *E. maxima* infection, thus providing a favorable environment for chicken growth. The underlying mechanism of action of β-alanine in suppressing *E. maxima*-induced inflammatory cytokine response needs further study but may involve a new protein synthesis that counteracts local inflammatory responses caused by infection. This means that the nutrients that should be used for growth could be wasted in producing proteins to control immune responses [43,44].

In general, inflammatory responses can damage the normal function and structure of tight junction proteins in the intestine [45]. In particular, the emergence of pro-inflammatory cytokines such as TNF-α and IL-1β is known to weaken the integrity of tight junctions [46]. However, in the current experiment, only Claudin-2 showed a tendency to decrease due to *E. maxima* infection, but even this did not have statistical significance. Other tight junction proteins showed no statistical difference despite *E. maxima* infection. These results may be related to the degree of intestinal damage mediated by coccidiosis and the dose of *E. maxima* oocysts used to induce coccidiosis. The degree of infection is a crucial factor in determining the success of animal experiments using infectious agents. If the infection is a weak one, it will be difficult to show the benefits of dietary supplementation but if the level of infection is too strong, it causes severe damage, leading to death without recovery. In the current experiment, infection was mild with minimal effect on cytokine responses and had no effect on TJ proteins. β-alanine, however, showed efficacy in enhancing JAM-2 and occludin. These results suggest that beta-alanine can activate specific protein phosphorylation pathways, such as the mammalian target of rapamycin (mTOR), thereby increasing the expression of TJ proteins like JAM-2 and occludin [47,48,49].

In the current experiment, 10 nutrient transporters were measured in the jejunum, with most of them decreased following *E. maxima* infection due to the gut damage caused by the intracellular development of *E. maxima* in the jejunal epithelial cells. This parasite-induced gut damage could possibly lead to a decrease in the gene expression level related to nutrient transporters [50]. A similar decrease in the gene expression of many nutrient transporters due to Eimeria infection has been reported in previous research reports [40,42]. In the current experiment, we showed the decreased expression of BAT1, B0AT1, LAT1, and LAT2, which are transporters related to amino acid absorption or reabsorption. Damage to these transporters could potentially hinder effective amino acid absorption. Interestingly, dietary β-alanine supplementation significantly increased the gene expression levels of BAT1, B0AT1, LAT1, and LAT2, which resulted from *E. maxima* infection. Importantly, all of these are transporters related to amino acid absorption or reabsorption. Furthermore, BAT1 and B0AT1 are primarily located in the apical part of the jejunum epithelial cells [51], and LAT2 is primarily located in the basolateral part [52]. However, further research will be necessary to understand how these transporters are interconnected to produce such results.

In an animal challenge study, *E. maxima* infection resulted in gut damage with increased gut lesion scores and oocyst number in the jejunum of infected chickens. This likely led to the onset of jejunal inflammatory responses, damaged gut transporters necessary for amino acid absorption, and, collectively, reduced the growth of chickens. Dietary β-alanine in *E. maxima*-infected chickens provided beneficial effects on gut health and mitigated the detrimental effects of *Eimeria* infection.

## 5. Conclusions

In conclusion, both in vitro and in vivo studies support the notion that dietary β-alanine mitigates LPS-induced inflammatory responses in chicken macrophage cells and local intestinal inflammatory response in *E. maxima*-infected chickens. Dietary β-alanine, in young chickens from the hatch, effectively strengthened gut integrity, mitigated *Eimeria*-induced intestinal damage, and reduced parasite fecundity, thereby reducing coccidiosis-induced growth retardation in broiler chickens infected with *E. maxima*. This study demonstrates the beneficial effects of dietary supplementation of young chickens with gut metabolites such as β-alanine, which was identified through global metabolomics, and further indicates the potential application of beneficial gut metabolites as novel feed additives to reduce the use of growth-promoting antibiotics to mitigate the negative effects of devastating parasitic infection such as coccidiosis in young broiler chickens.

## Figures and Tables

**Table 1:** Sequences of oligonucleotide primer used for qRT-PCR.

Type	Target Gene	Samples	Primer Sequence (5′-3′)	Accession Number or References
Reference	GAPDH		F-GGTGGTGCTAAGCGTGTTAT	K01458
			R-ACCTCTGCCATCTCTCCACA	
Proinflammatory	IL-1β	CMCs and Jejunum	F-TGGGCATCAAGGGCTACA	Y15006
			R-TCGGGTTGGTTGGTGATG	
	IL-6	CMCs and Jejunum	F-CAAGGTGACGGAGGAGGAC	AJ309540
			R-TGGCGAGGAGGGATTTCT	
	IL-8	CMCs and Jejunum	F-GGCTTGCTAGGGGAAATGA	AJ009800
			R-AGCTGACTCTGACTAGGAAACTGT	
	TNFSF15	Jejunum	F-CCTGAGTATTCCAGCAACGCA	AB194710
			R-ATCCACCAGCTTGATGTCACTAAC	
Th	IFN-γ	Jejunum	F-AGCTGACGGTGGACCTATTATT	NM_205149
			R-GGCTTTGCGCTGGATTC	
	IL-10	Jejunum	F-CGGGAGCTGAGGGTGAA	AJ621614
			R-GTGAAGAAGCGGTGACAGC	
TJ proteins	Claudin-1	Jejunum	F-TGGAGGATGACCAGGTGAAGA	NM_001013611.2
			R-CGAGCCACTCTGTTGCCATA	
	Claudin-2	Jejunum	F-CCTGCTCACCCTCATTGGAG	NM_001277622.1
			R-GCTGAACTCACTCTTGGGCT	
	JAM-2	Jejunum	F-AGCCTCAAATGGGATTGGATT	NM_001006257.1
			R-CATCAACTTGCATTCGCTTCA	
	Occludin	IECs and jejunum	F-GAGCCCAGACTACCAAAGCAA	NM205,128.1
			R-GCTTGATGTGGAAGAGCTTGTTG	
	ZO-1	IECs and jejunum	F-CCGCAGTCGTTCACGATCT	XM01,527,8981.1
			R-GGAGAATGTCTGGAATGGTCTGA	
	ZO-2	Jejunum	F-ATCCAAGAAGGCACCTCAGC	NM_204918.1
			R-CATCCTCCCGAACAATGC	
Mucin	MUC2	IECs	F: GCCTGCCCAGGAAATCAAG	NM0,013,18434.1
			R: CGACAAGTTTGCTGGCACAT	
Muscle cell	*MyoG*	QMCs and PMCs	F-CTGCCCAAGGTGGAGATCCT	Choi et al. [30]
			R-CTGGAGTTTGGCACCAACCC	
	*Pax7*	QMCs and PMCs	F- TCGATTAGCCGTGTGCTACG	NM_205065.1
			R-GCCATCTATGCTGTGCTTGG	
Nutrient transports	BAT1	Jejunum	F-GGGTTTTGTGTTGGCTTAGGAA	XM_419056.6
			R-TCCATGGCTCTGGCAGAGAT	
	B0AT	Jejunum	F-CAGTAGTGAATTCTCTGAGTGTGAAGCT	NM_001199133.1
			R-GCAATGATTGCCACAACTACCA	
	CAT1	Jejunum	F-CCAAGCACGCTGATAAAG	XM_015277945.2
			R-TACTCACAATAGGAAGAAGGG	
	EAAT	Jejunum	F-TGCTGCTTTGGATTCCAGTGT	XM_424930.6
			R-AGCAATGACTGTAGTGCAGAAGTAATATATG	
	GLUT1	Jejunum	F-CTTTGTCAACCGCTTTGG	NM_205209.1
			R-TGTGCCCCGGAGCTTCT	
	GLUT2	Jejunum	F-TCATTGTAGCTGAGCTGTT	NM_207178.1
			R-CGAAGACAACGAACACATAC	
	GLUT5	Jejunum	F-TTGCTGGCTTTGGGTTGTG	XM_417596.6
			R-GGAGGTTGAGGGCCAAAGTC	
	LAT1	Jejunum	F-GATTGCAACGGGTGATGTGA	NM_001030579.2
			R-CCCCACACCCACTTTTGTTT	
	LAT2	Jejunum	F-TCAGCTTCAGTTACTGGTT	XM_025154295.1
			R-GCACAACCACGAGAAATAC	
	SGLT	Jejunum	F-GCCGTGGCCAGGGCTTA	NM_001293240.1
			R-CAATAACCTGATCTGTGCACCAGT	

CMCs: chicken macrophage cells, IECs: chicken intestinal epithelial cells, QMCs: quail muscle cells, PMCs: primary chicken embryonic muscle cells, Th: T helper cells, TJ: tight junction, BAT1: Na^+^-dependent amino acid transporter, B0AT1: Na^+^-independent amino acid transporter, CAT1: cationic amino acid transporter, EAAT: excitatory amino acid transporter, GLUT1: glucose transporter 1, GLUT2: glucose transporter 2, GLUT5: glucose transporter 5, LAT1: L-type amino acid transporter 1, LAT2: Na^+^-dependent neutral/cationic amino acid transporter, *MyoG*: Myogenin, *Pax7*: Paired Box 7, SGLT: sodium–glucose transporter.

**Table 2:** Composition of basal diet ingredients (as-fed basis, %, unless otherwise specified).

Ingredients (%)	Basal Diet
Corn	55.78
Soybean meal	37.03
Soybean oil	2.97
Dicalcium phosphate	1.80
Calcium carbonate	1.51
Salt	0.38
Poultry Vit Mix ^1^	0.22
Poultry Mineral Mix ^2^	0.15
DL-Methionine	0.10
Choline-chloride, 60%	0.06
Total	100
Calculated values (%)	
CP, %	24.00
Ca, %	1.20
AP, %	0.51
Lys, %	1.40
Met, %	0.49
Cys + Met, %	0.80
ME, Mcal/kg	3.5

^1^ Vitamin mixture per kg of diet: vitamin A, 2000 IU; vitamin D3, 22 IU; vitamin E, 16 mg; vitamin K, 0.1 mg; vitamin B_1_, 3.4 mg; vitamin B_2_, 1.8 mg; vitamin B_6_, 6.4 mg; vitamin B_12_, 0.013 mg; biotin, 0.17 mg; pantothenic acid, 8.7 mg; folic acid, 0.8 mg; niacin, 23.8 mg; ^2^ Mineral mixture per kg of diet: Fe, 400 mg; Zn, 220 mg; Mn, 180 mg; Co, 1.3 mg; Cu, 21 mg; Se, 0.2 mg. CP: crude protein, AP: available phosphorus.

**Table 3:** Body weight and average daily gain of chickens fed a β-alanine supplemented diet during *Eimeria maxima* infection.

Treatment	NC	PC	H-ALA	L-ALA	SEM	*p*-Value
BW, g						
Initial	36.6	36.6	36.6	36.6	0.7	1.000
d 7	157	159	160	160	2.5	0.822
d 14 (0 dpi)	461	457	464	475	8.1	0.448
d 20 (6 dpi)	860 ^a^	743 ^b^	738 ^b^	764 ^b^	16	<0.001
d 22 (8 dpi)	1017 ^a^	763 ^c^	843 ^b^	839 ^b^	22	<0.001
ADG, g						
d 0 to 7	20.0	20.4	20.6	20.6	0.3	0.475
d 7 to 14	43.5	41.9	43.5	45.1	0.9	0.109
d 0 to 14 ^1^	32.7	32.2	32.9	33.8	0.6	0.291
d 14 to 20	66.5 ^a^	47.7 ^b^	45.5 ^b^	47.2 ^b^	1.6	<0.001
d 14 to 22 ^2^	69.4 ^a^	38.2 ^c^	47.3 ^b^	43.3 ^b^	1.7	<0.001

NC: basal diet, PC: basal diet for *E. maxima*-infected chickens, H-ALA: β-alanine at 10.0 mg/kg feed, L-ALA: β-alanine at 1.0 mg/kg feed, SEM: standard error of the mean, ^1^ before infection, ^2^ entire infection period, ADG: average daily gain, BW: body weight, d: day, dpi: days post infection, all chickens, except NC, were infected by oral gavage on day 14 with 1.0 × 10^4^ oocysts/chicken of *E. maxima*. ^a~c^ Means in the same row with different superscripts differ (*p* < 0.05), and the difference was evaluated using the PDIFF option in SAS when the *p*-value between treatments was less than 0.05.

**Table 4:** The expression level of jejunal nutrient transporter genes in chickens fed a β-alanine supplemented diet during *E. maxima* infection.

	NC	PC	H-ALA	L-ALA	SEM	*p*-Value
Nutrient transporters, mean normalized expression						
BAT1	0.43 ^a^	0.13 ^c^	0.29 ^b^	0.16 ^c^	0.04	0.001
B0AT1	0.074 ^a^	0.027 ^c^	0.053 ^ab^	0.035 ^bc^	0.009	0.008
CAT1	0.031	0.016	0.033	0.021	0.008	0.380
EAAT	0.041	0.034	0.034	0.035	0.009	0.940
GLUT1	0.0057 ^a^	0.0013 ^b^	0.0033 ^ab^	0.0014 ^b^	0.001	0.011
GLUT2	0.022 ^a^	0.007 ^b^	0.008 ^b^	0.008 ^b^	0.004	0.023
GLUT5	0.073 ^a^	0.016 ^b^	0.038 ^b^	0.035 ^b^	0.010	0.007
LAT1	0.070 ^a^	0.015 ^c^	0.05 ^ab^	0.03 ^bc^	0.009	0.004
LAT2	0.0072 ^a^	0.0014 ^b^	0.0050 ^a^	0.0018 ^b^	0.001	0.001
SGLT	0.14	0.11	0.12	0.12	0.03	0.963

NC: basal diet, PC: basal diet for infected chickens, H-ALA: β-alanine at 10.0 mg/kg feed, L-ALA: β-alanine at 1.0 mg/kg feed, BAT1: Na^+^-dependent amino acid transporter, B0AT1: Na^+^-independent amino acid transporter, CAT1: cationic amino acid transporter, EAAT: excitatory amino acid transporter, GLUT1: glucose transporter 1, GLUT2: glucose transporter 2, GLUT5: glucose transporter 5, LAT1: L-type amino acid transporter 1, LAT2: Na^+^-dependent neutral/cationic amino acid transporter, SGLT: sodium–glucose transporter. All chickens, except the NC, were infected via oral gavage at day 14 with 1.0 × 10^4^ oocysts/chicken of *E. maxima*. ^a~c^ Means in the same row with different superscripts differ (*p* < 0.05), and the difference was evaluated using the PDIFF option in SAS when the *p*-value between treatments was less than 0.05. Data (*n* = 6) were collected at day 20 (6 dpi: days post infection). Transcript levels of the jejunal nutrient transporters were measured using quantitative RT-PCR and normalized to GAPDH levels.

## Data Availability

The data presented in this study are available from the corresponding author upon request.
